# Linearized esculentin-2EM shows pH dependent antibacterial activity with an alkaline optimum

**DOI:** 10.1007/s11010-021-04181-7

**Published:** 2021-06-06

**Authors:** Erum Malik, David A. Phoenix, Timothy J. Snape, Frederick Harris, Jaipaul Singh, Leslie H. G. Morton, Sarah R. Dennison

**Affiliations:** 1 College of Medicine and Health, University of Exeter Medical School, Heavitree Road, Exeter, EX1 2LU UK; 2 Office of the Vice Chancellor, London South Bank University, 103 Borough Road, London, SE1 0AA UK; 3 School of Pharmacy, De Montfort University, Leicester, LE1 9BH UK; 4 School of Natural Sciences, University of Central Lancashire, Preston, PR1 2HE UK; 5 School of Pharmacy and Biological Sciences, University of Central Lancashire, Preston, PR1 2HE UK

**Keywords:** Linearized esculentin 2EM (E2EM-lin), α-Helical structure, pH dependent with alkaline optimum, Tilted peptide, Preference for gram-positive bacteria

## Abstract

Here the hypothesis that linearized esculentin 2EM (E2EM-lin) from *Glandirana emeljanovi* possesses pH dependent activity is investigated. The peptide showed weak activity against Gram-negative bacteria (MLCs ≥ 75.0 μM) but potent efficacy towards Gram-positive bacteria (MLCs ≤ 6.25 μM). E2EM-lin adopted an α-helical structure in the presence of bacterial membranes that increased as pH was increased from 6 to 8 (↑ 15.5–26.9%), whilst similar increases in pH enhanced the ability of the peptide to penetrate (↑ 2.3–5.1 mN m^−**1**^) and lyse (↑ 15.1–32.5%) these membranes. Theoretical analysis predicted that this membranolytic mechanism involved a tilted segment, that increased along the α-helical long axis of E2EM-lin (1–23) in the N → C direction, with −  < *µH* > increasing overall from *circa* − 0.8 to − 0.3. In combination, these data showed that E2EM-lin killed bacteria via novel mechanisms that were enhanced by alkaline conditions and involved the formation of tilted and membranolytic, α-helical structure. The preference of E2EM-lin for Gram-positive bacteria over Gram-negative organisms was primarily driven by the superior ability of phosphatidylglycerol to induce α-helical structure in the peptide as compared to phosphatidylethanolamine. These data were used to generate a novel pore-forming model for the membranolytic activity of E2EM-lin, which would appear to be the first, major reported instance of pH dependent AMPs with alkaline optima using tilted structure to drive a pore-forming process. It is proposed that E2EM-lin has the potential for development to serve purposes ranging from therapeutic usage, such as chronic wound disinfection, to food preservation by killing food spoilage organisms.

**Supplementary Information:**

The online version contains supplementary material available at 10.1007/s11010-021-04181-7.

## Introduction

It is well established that pH plays an important physiological role in humans that is tightly regulated by acid–base homeostasis; however, unregulated changes in pH can impact on human health via multiple routes [1]. A number of disorders and diseases are associated with low pH [2–4] and a particularly important example is cystic fibrosis (CF), which is a progressive, genetic disease that is caused by mutations in the cystic fibrosis transmembrane conductance regulator gene [5, 6]. CF is characterized by a predisposition to persistent microbial infection that is believed to involve disrupted bicarbonate production, resulting in an abnormally acidic pH in the airway surface liquid of the lungs [7, 8]. These pH conditions appear to impair host airway defences through a number of mechanisms, including decreasing ciliary beat and reducing the efficacy of endogenous antimicrobials, thereby promoting dysbiosis of the airway microbiome and chronic infections [9–11]. In addition to low pH, a number of disorders and diseases are associated with high pH [2, 3, 12]; for example, pathological skin conditions such as psoriasis [13], acne [14] and atopic dermatitis [15]. In these cases, alkaline conditions are produced by the collaborative action of a variety of exogenous and endogenous factors, such as skin type and dysregulated skin buffering, respectively [16–18]. These pH conditions are believed to decrease the production and potency of endogenous antimicrobials, thereby inducing inflammation, dysbiosis of the skin microbiome and microbial colonization of the skin [19–22].

It is becoming increasingly clear that abnormal pH is not only associated with the genesis and progress of a number of diseases and disorders, but also presents difficulties in their treatment [23–25]. Abnormal pH is able to critically reduce the efficacy of many conventional antibiotics; for example, the potency of macrolides is decreased by acid conditions and that of β-lactams is diminished by alkaline conditions [23, 26, 27]. The need for antibiotics to treat pH related diseases and disorders has been further exacerbated by the general lack of novel antibiotics available, with those under development generally produced by the modification of existing classes of these molecules. [28–30]. In response, there have been concerted attempts to identify new antibiotics with novel mechanisms of action [31–33] and an attractive proposition is the development of antimicrobial peptides (AMPs), which are endogenous defence molecules produced by organisms across the eukaryotic kingdom [30, 34]. AMPs with pH dependent activity and acid optima have been identified in wide variety of creatures ranging from cattle and rodents to mollusks and nematodes, and a number of these peptides have been developed for medical and biotechnical uses [9]. As a recent example, GKY25 and HVF18, are thrombin-derived AMPs that were identified in wound fluid and were found to have potent antibacterial activity at the acidic pH of healthy skin, which led to the proposal that these peptides could potentially be developed as topical or systemic antimicrobial agents [35]. However, in contrast, pH dependent AMPs with alkaline optima have been described in relatively few creatures, to date: humans, rabbits, sheep, fish and amphibians are generally less well characterized than AMPs with acid optima [36]. In response, the present study investigates the hypothesis that the linearized form of esculentin 2EM (E2EM-lin) from *Glandirana emeljanovi* (The Imienpo Station frog) possesses pH dependent, antibacterial activity. These investigations show that the peptide has potent membranolytic action against Gram-positive bacteria that is enhanced by alkaline pH, and it is proposed that E2EM-lin has the potential for development to serve purposes ranging from therapeutic usage to food protection. In addition, insights gained from structure function/relationships identified as underpinning the membrane interactions and antibacterial activity of E2EM-lin have been used to update a model recently proposed to explain the antimicrobial activity of the peptide [37].

## Materials and methods

### Materials

E2EM-lin (GILDTLKQFAKGVGKDLVKGAAQGVLSTVSCKLAKTC) was supplied by Pepceuticals (UK), synthesised by solid phase synthesis and purified by HPLC to purity greater than 99%, confirmed by MALDI mass spectrometry. Sodium phosphate monobasic, sodium diphosphate dibasic, Sephadex G75, HEPES [4-(2-hydroxyethyl)-1-piperazineethanesulfonic acid] and EDTA (Ethylenediaminetetraacetic acid), sodium chloride and hydrochloric acid were supplied by Sigma-Aldrich Ltd (UK). Sodium hydroxide was purchased from BDH laboratory supplies, pH indicators strips were obtained from VWR (UK) whilst Triton X-100 and chloroform were supplied by Thermo Fisher Scientific (UK). Dimyristoyl phosphatidylglycerol (DMPG), dimyristoyl phosphatidyl ethanolamine (DMPE), and cardiolipin (CL) were supplied by Avanti Polar Lipids, Inc., and Calcein was supplied by Alfa Aesar. Milli Q water with a specific resistance of 18 Ω cm^−1^ was used for preparation of stock solutions and buffers.

### Methods

#### The antibacterial properties of E2EM-lin

The ability of E2EM-lin to kill *Staphylococcus aureus* (strain NCIMB 6571), *Bacillus subtilis* (strain NCIMB 1671), *Escherichia coli* (strain W3110) and *Pseudomonas aeruginosa* (strain NCIMB 10,848) was assayed (Table 1). Cell suspensions of these bacteria were prepared using freeze–dried cultures grown on Nutrient agar to inoculate a series of 9 ml of sterile Nutrient broths in universal bottles. These samples were then incubated in an orbital shaker at 100 rpm and 37 °C until reaching their exponential log phase, as determined by optical density measurements in the range 0.01–0.03 at λ = 600 nm. Each bacterial suspension was centrifuged at 15,000 × *g* for 15 min at 21 °C using a bench top centrifuge (ALC PK 120R) to form a cell pellet. The resulting cell pellets were resuspended in 9 ml Ringer’s solution and centrifuged again, and the resulting pellets were resuspended in Ringer’s solution to give a starting inoculum density of *circa* 5.8 × 10^8^ CFU/ml. In order to evaluate the toxicity of E2EM-lin to bacterial cells, stock peptide in 25% Ringer’s solution (150 μM), was diluted to give concentrations in the range 0.06–150 μM. Aliquots (500 μl) of the peptide at each concentration in this range were then inoculated with an equal amount of bacterial suspension (10 μl) and left to incubate for 12 h at 37 °C. As a control, this procedure was repeated without the inclusion of E2EM-lin. After incubation, each sample of was spread onto a Nutrient agar plate and incubated for 12 h to determine the minimum peptide concentration that yielded no bacterial growth. Where no bacterial growth was observed, 10 μl samples of these E2EM–bacteria mixtures were used to inoculate 9.9 ml of fresh Nutrient broth and incubated for a further 12 h. After incubation, these peptide–bacterial mixtures were spread onto Nutrient agar plates and where no bacterial growth had occurred, the levels of E2EM-ln in that sample was taken as its minimum lethal concentration (MLC) for the organism concerned. These experiments were performed in quintuplicate and the mean value derived.

**Table 1:** Major AMPs with pH dependent antimicrobial activity and alkaline optima

AMPs	Source organism
MCP-1 and MCP-2	Rabbits
Tβ-4	Humans
MUC7 12-mer peptide	Humans
SMAP29	Sheep
OaBac5mini,	Sheep
OaBac7.5mini	Sheep
Salmine (protamine)	*Salmo salar*
Clupeine (protamine)	*Clupea harengus*
Clupeine (protamine)	*Clupea pallasii*
Dy2	*Rana dybowskii*
AWRK6	*Rana dybowskii*
FL9	*Leptodactylus fallax*

This table was compiled from [36]

#### The potential of E2EM-lin for tilted peptide formation

The potential for tilted membrane interaction by E2EM-lin was analyzed according to extended hydrophobic moment methodology [38], which uses the mean hydrophobic moment,  < *µ*_*H*_ *>* to quantify the amphiphilicity of a α-helical sequence, and the mean hydrophobicity, < *H* *>* , of the sequence to measure its affinity for the membrane interior [39]. For this analysis, *<µ*_*H*_ *>* and  < *H* *>* were computed using the normalised consensus hydrophobicity scale of Eisenberg et al., (1982) and a moving window of 11 residues [38]. The values of < *µH* > and < *H* > obtained were then plotted on the extended hydrophobic moment plot diagram, which identifies candidate tilted α-helix forming segments [38]. The potential of E2EM-lin to form hydrophobicity gradients, which is characteristic of tilted α-helical architecture, was visualized by amphiphilic profiling using − < *µH* > with a moving window of seven residues and the normalized consensus hydrophobicity scale of Eisenberg et al., (1982) [40]. The net charge on E2EM-lin was determined using the software at (http://pepcalc.com/) and a visual representation of the potential tilted structure formed by E2EM-lin was obtained using the graphics function of Heliquest software to generate two-dimensional axial projections of these peptides (available online at http://heliquest.ipmc.cnrs.fr/) [41].

#### The effect of pH on the conformation of E2EM-lin in the presence of bacterial membranes

The conformational behaviour of E2EM-lin in aqueous solution was investigated by solubilising the peptide in PBS (10 mM) at either pH 6, 7 or 8, to give a final peptide concentration of 2.6 μM. The conformational behaviour of E2EM-lin in the presence of a range of small unilamellar vesicles (SUVs) was investigated by solubilising the peptide (final concentration of 0.1 mg ml^−1^) in these SUVs at a lipid to peptide ratio of 1:100. SUVs were formed from either DMPG, CL or DMPE, which were taken to represent the major lipids in bacterial membranes, namely: species of phosphatidylglycerol (PG), cardiolipin (CL) and phosphatidylethanolamine (PE). SUVs were also formed from lipid mixtures mimetic of membranes from *B. subtilis*, *S. aureus*, *E. coli* and *P. aeruginosa* (Table 2). In each case, these single lipids and lipid mixtures were dissolved separately in chloroform and dried under N_2_ gas before being placed under vacuum for 4 h. The resulting lipid films were rehydrated using PBS (10 mM) at either pH 6, 7 or 8 and then sonicated for an hour or until the solution was no longer turbid, after which samples were subjected to five cycles of freeze-thawing. Conformational analyses were conducted using a J-815 CD spectropolarimeter (Jasco, UK) at 20 °C, all as previously described [42]. Essentially, samples were placed in a quartz cell with a 10 mm path-length and four scans per sample were performed over a wavelength range of 260–180 nm at 0.5 nm intervals, using a band width of 2 nm and a scan speed 50 nm min^−1^. All spectra were baseline corrected and the % α-helical content determined using the CDSSTR method (protein reference set 3) from the DichroWeb server [43–45]. These experiments were repeated in quintuplicate and the levels of α-helicity obtained were averaged.

**Table 2:** The lipid composition of bacterial membranes and the antibacterial activity of E2EM-lin

Test organisms	Membrane lipid composition and molar ratio	E2EM-lin MLCs (µM)
Gram-positive bacteria		
* Staphylococcus aureus*	DMPG:CL (57:43)	3.13
* Bacillus subtilis*	DMPE:DMPG:CL (10:29:47)	6.25
Gram-negative bacteria		
* Escherichia coli*	DMPE:DMPG:CL (82:6:12)	100.00
* Pseudomonas aeruginosa*	DMPE:DMPG:CL (68:19:11)	75.00

In this table, DMPG dimyristoyl phosphatidylglycerol, DMPE dimyristoyl phosphatidylethanolamine and CL cardiolipin, and data for the lipid compositions of bacterial membranes were derived from [55, 56]. MLCs are the minimum lethal concentrations of E2EM-lin required to kill bacteria and the data are the mean of five replicates

#### The effect of pH on the ability of E2EM-lin to penetrate and lyse bacterial membranes

The ability of E2EM-lin to penetrate bacterial membranes was determined using a 601 M Langmuir trough (Biolin Scientific KSV NIMA, UK) equipped with moveable barriers. Surface pressure changes were monitored using a Whatman CH1 Wilhelmy paper plate attached to a microbalance [46, 47]. In all experiments the subphase of the Langmuir trough consisted of PBS buffer (10 mM) at the appropriate pH, which was prepared with Milli-Q-water (resistivity≈18 MΩ cm) at 21 ± 1 °C.

The effect of pH on the ability of E2EM-lin to penetrate lipid monolayers was studied at constant area by separately spreading chloroformic solutions onto a subphase of PBS (10 mM), at either pH 6, 7 or 8. which contained either the individual lipids DMPG, CL or DMPE, or lipid mixtures mimetic of membranes from *S. aureus*, *B. subtilis*, *E. coli* and *P. aeruginosa* (Table 2). After spreading these monolayers, solvent was allowed to evaporate for 30 min and then the barriers Langmuir trough were closed at a rate of 10 cm^2^ min^−1^ to achieve a surface pressure of 30 mN m^−1^. This surface pressure is generally taken to represent the packing density of naturally occurring cell membranes and was maintained throughout these experiments [48]. Monolayers were allowed to equilibrate for 10 min and E2EM-lin was injected into the subphase to give a final peptide concentration of 0.5 μM. The resulting surface pressure increases were monitored and plotted as a function of time. Each of these experiments was performed in quintuplicate and the mean value derived.

The effect of pH on the ability of E2EM-lin to lyse bacterial membranes was investigated using chloroformic solutions, which contained either the individual lipids DMPG, CL or DMPE, or lipid mimics of membranes from *S. aureus*, *B. subtilis*, *E. coli* and *P. aeruginosa* (Table 2). These chloroformic lipid solutions were then used to form SUVs and were dried under an N_2_ (gas) stream before being placed under vacuum overnight. The resulting thin lipid films were hydrated using 5.0 mM HEPES, which contained 70 mM calcein, and these suspensions were then vortexed before being sonicated for 30 min and freeze-thawed 5 times. Untrapped calcein was separated from dye filled SUVs by gel filtration using a Sephadex G75 column, which was rehydrated overnight in 20 mM HEPES, 150 mM NaCl and 1.0 mM EDTA. The column was eluted with 5 mM HEPES pH 7.5 to produce solutions of SUVs with calcein entrapped. The rate of calcein leakage induced by E2EM-lin from these SUVs was then determined as a function of peptide concentration. Stock solutions of E2EM-lin (90 µl) with concentrations in the range, 0–200 µM, were mixed with 30 µl of calcein entrapped SUVs solutions and these samples made up to 3 ml with PBS, with PBS (10 mM) at either pH 6, 7 or 8. The samples were left to incubate and after one hour, calcein fluorescence was measured using an FP-6500 spectrofluorometer (JASCO, UK) with an excitation wavelength of 490 nm and an emission wavelength of 520 nm. The % calcein leakage from SUVs was then calculated according to Eq. 1:

1 $$ \% {\text{ dye release}} = \left( {\left[ {{\text{F}}_{{{\text{Peptide}}}} } \right] - \left[ {{\text{F}}_{{{\text{PBS}}}} } \right]} \right)/\left( {\left[ {{\text{F}}_{{{\text{Triton}}}} } \right] - \left[ {{\text{F}}_{{{\text{PBS}}}} } \right]} \right) \times {1}00 $$

where the fluorescence of calcein release by the peptide at 520 nm is denoted by [F_Peptide_], that released by PBS as [F_PBS_] and that released by Triton X-100 as [F_Triton_]. In all cases, values of the % calcein released were determined in quintuplicate and the mean value derived.

#### The effect of pH on the thermodynamic stability of E2EM-lin interactions with bacterial membranes

The thermodynamic stability of E2EM-lin interactions with monolayers mimetic of bacterial membranes was studied using a 601 M Langmuir trough (Biolin Scientific KSV NIMA, UK) equipped with moveable barriers. Surface pressure changes were monitored using a Whatman CH1 Wilhelmy paper plate attached to a microbalance [46, 47]. In all experiments the subphase of the Langmuir trough consisted of PBS buffer (10 mM) at the appropriate pH, which was prepared with Milli-Q-water (resistivity≈18 MΩ cm) at 21 ± 1 °C.

Compression isotherms were generated by spreading chloroformic solutions of lipid (1.0 × 10^15^ molecules) onto a subphase of PBS (10 mM) at either pH 6, 7 or 8, which contained lipid mixtures mimetic of membranes from *S. aureus*, *B. subtilis*, *E. coli* and *P. aeruginosa* (Table 2). The solvent was allowed to evaporate for 10 min and the monolayer was allowed to settle for a further 20 min before the trough barriers were closed at a rate of 10 cm^2^ per minute, until monolayer collapse pressure was achieved. Surface pressure changes were monitored and plotted as a function of the area per lipid molecule. Corresponding experiments were then performed except that E2EM-lin was injected into the PBS subphase to give a final peptide concentration of 2 µM_._ All experiments were carried out at room temperature, 25 °C, repeated in quintuplicate and the mean value derived.

Thermodynamic analysis of lipid and lipid / peptide isotherms was undertaken and the thermodynamic stability of these isotherms was investigated by determining their Gibbs free energy of mixing (Δ*G*_*mix*_) according to Eq. (2):

2 $$ \Delta G_{{{\text{mix}}}} = \int {\left[ {A_{1,2,3} - \left( {X_{1} A_{1} + X_{2} A_{2} + X_{3} A_{3} } \right)d\pi } \right]} $$

where *A*_1,2,3*.*_is the molecular area occupied by the mixed monolayer, *A*_1_–*A*_3_ are the area per lipid molecule in the pure monolayers of component 1, 2, and 3, *X*_1_, *X*_2_, *X*_3_ are the molar fractions of the components. Numerical data were calculated from these compression isotherms according to the mathematical method of Simpson [49]. Each of these experiments was performed in quintuplicate and the mean value derived. Δ*G*_mix_ is used to measure the relative stability of monolayers associated with the miscibility energetics of their pure lipid components where thermodynamically stable and unstable monolayers are indicated by negative and positive values of Δ*G*_mix_, respectively [50, 51].

#### Statistical analysis

Unless otherwise stated, the results are presented as mean values ± standard errors (SE) of the mean. Initially, the normal distribution of the data was analysed by Skewness Kurtosis tests. Significant differences between mean values were further analysed using a one-way ANOVA test, based on a null hypothesis that there is no significant difference between the mean values.

## Results

### The antibacterial activity of E2EM-lin

Antibacterial assay of E2EM-lin showed that the peptide had weak activity against the Gram-negative organisms, *E. coli* and *P. aeruginosa*, with MLCs that were ≥ 75.0 μM, but possessed potent efficacy towards the Gram-positive bacteria, *S. aureus* and *B. subtilis*, with MLCs that were ≤ 6.25 μM (Table 2). Statistical analysis showed that there was a significant difference between the MLCs of E2EM-lin for these Gram-negative and Gram-positive bacteria (F_3_, _11_ = 74,217; *p* = 0.000), and in combination, these observations indicated that the peptide has a strong preference for Gram-positive bacteria, which is consistent with data reported by previous authors [52].

### The potential of E2EM-lin for tilted peptide formation

**Fig. 1:**
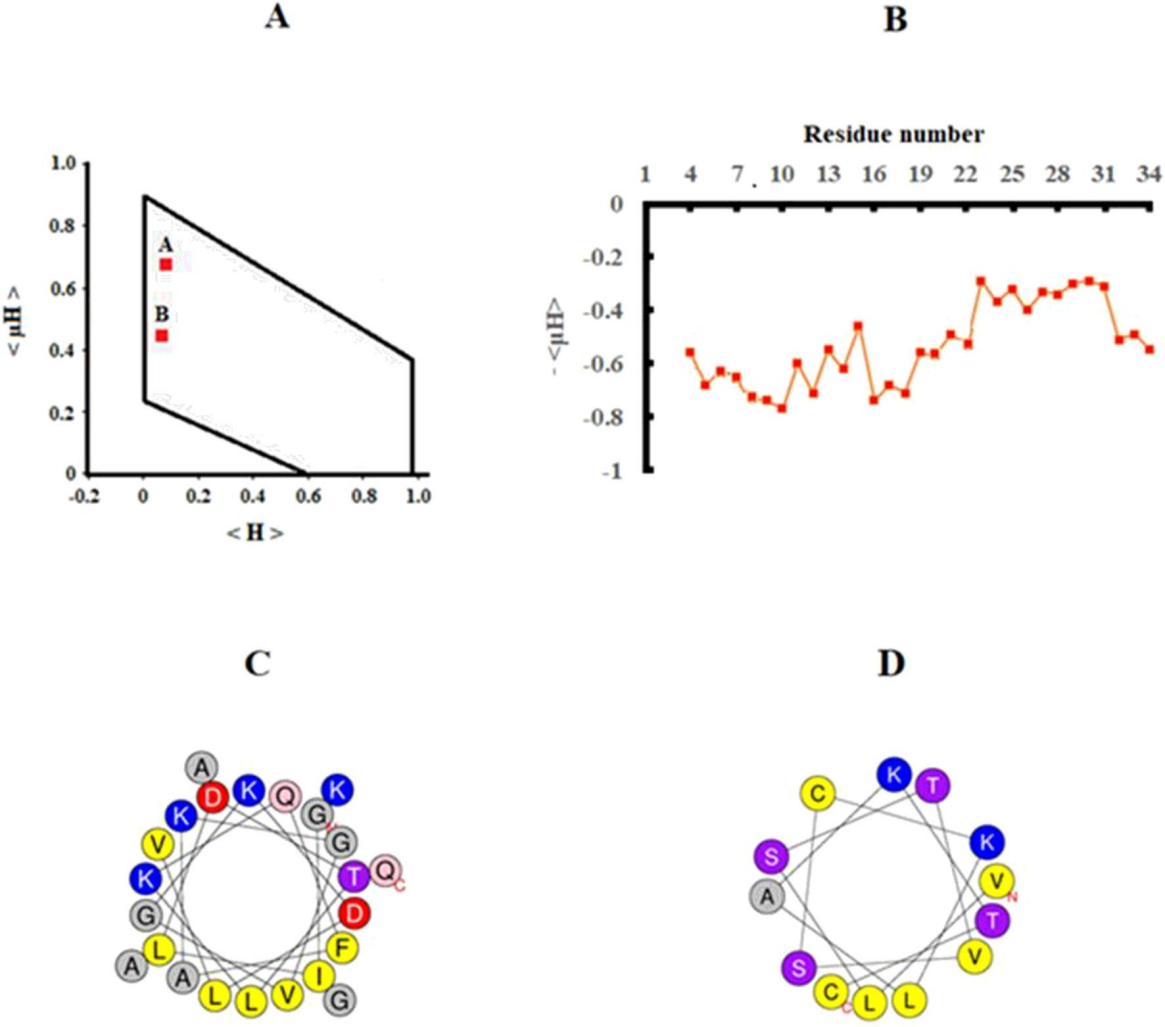
The potential of AMPs for tilted peptide formation. In **A**, extended hydrophobic moment plot methodology showed that the data points representing E2EM-lin (1–23) [red square (A), < *µH* > = 0.67 and < *H* > = 0.09] and E2EM-lin (25–37) [red square (B), < *µH* > = 0.43 and < *H*   = 0.06] lay in the shaded area of the plot diagram, indicating the potential to form tilted peptide structure (**A**). In **B**, amphiphilic profiling revealed that E2EM-lin (1–23) [red line (A)] possessed a hydrophobicity gradient along the α-helical long axis, which is characteristic of tilted peptides [40, 54]. The hydrophobicity gradient formed by E2EM-lin (1–23) increased along the α-helical long axis in the N → C direction, with − < *µH* > increasing overall from *circa* − 0.8 to − 0.3 (**B**). In contrast, the amphiphilic profiling of E2EM-lin (25–37) showed that this segment formed no discernable hydrophobicity gradient over residues 25–37, which suggested that this region does not form tilted structure (**B**). In **C**, E2EM-lin (1–23) was modelled as a two-dimensional axial projection, which revealed that this α-helix possessed a hydrophobic face of *circa* 180° and a hydrophilic face, which is rich in lysine, aspartic acid and glycine residues (**C**). In **D**, E2EM-lin (25–37) was also modelled as a two-dimensional axial projection and was found to form a α-helix, possessing a hydrophobic face of 120° and a hydrophilic face rich in polar residues and lysine residues (**D**). (Color figure online)

E2EM-lin, forms an N-terminal, α-helical segment, E2EM-lin (1–23) and a shorter C-terminal, α-helical segment, E2EM-lin (25–37), that are separated by an intervening glycine residue at sequence position 24 (G24) [37, 53]. Extended hydrophobic moment plot analysis showed that the data points representing E2EM-lin (1–23) ( < *µH* > = 0.67,  < *H* > = 0.09) and E2EM-lin (25–37) (< *µH* > = 0.43 and < *H* > = 0.06) lay in the shaded area of the plot diagram, indicating the potential to form tilted structure (Fig. 1A). Amphiphilic profiling revealed that E2EM-lin (1–23) possessed a hydrophobicity gradient, which is an asymmetric distribution of hydrophobicity along the α-helical long axis that drives the parent molecule to penetrate membranes at a shallow angle of between 20° and 80° [40, 54]. The hydrophobicity gradient formed by E2EM-lin (1–23) increased along the α-helical long axis in the N → C direction, with − < *µH* > increasing from *circa* − 0.8 to − 0.3 (Fig. 1B). In contrast, the amphiphilic profiling of E2EM-lin (25–37) showed that this segment formed no discernable hydrophobicity gradient over residues 25–37, which suggested that this region does not form tilted structure (Fig. 1B). The net charge on E2EM-lin was determined using the software at (http://pepcalc.com/) which showed that this charge dropped from + 4 at acid pH to + 3 at alkaline pH, and two-dimensional axial projections were generated for E2EM-lin (1–23) and E2EM-lin (25–37) to provide visual representation of the potential tilted structure formed by these segments (Fig. 1C, D). E2EM-lin (1–23) formed a strongly amphiphilic α-helix (< *µH* > = 0.67), with a hydrophobic face of *circa* 180° and a hydrophilic face that included multiple aspartic acid residues and lysine residues (Fig. 1C). In contrast, E2EM-lin (25–37) formed a α-helix with lower amphiphilicity (< *µH* >  = 0.43) than that of E2EM-lin (25–37) that possessed a hydrophobic face of 120*°* and a hydrophilic face rich in polar residues and lysine residues (Fig. 1D).

### The effect of pH on the conformation of E2EM-lin in the presence of bacterial membranes

**Fig. 2:**
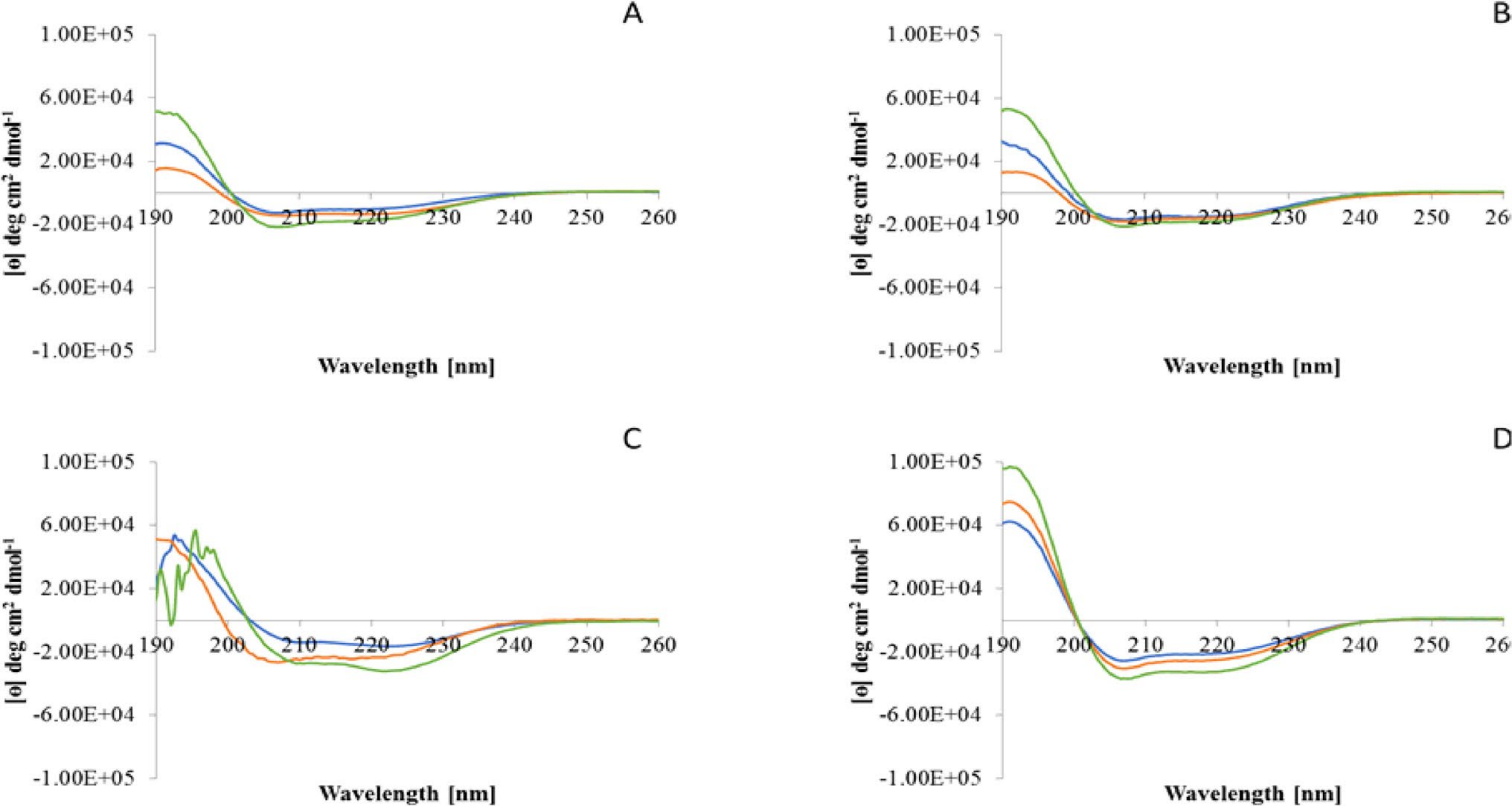
The effect of pH on the conformation of E2EM-lin in the presence of bacterial membranes. This figure shows the effect of changing pH on the conformational behaviour of E2EM-lin in the presence of SUVs mimetic of bacterial membranes, which are those representing *E. coli* (**A**), *P. aeruginosa* (**B**), *B. subtilis* (**C**) and *S. aureus* (**D**) at pH 6 (Blue), pH 7 (Orange) and pH 8 (Green). In all cases, these curves possesses minima in the range 210–224 nm and a maxim around 193 nm, which is typical of α-helical structure [42]. Analysis of these spectra showed that the levels of α-helicity possessed by E2EM-lin was enhanced as pH increased from pH 6 to pH 8 in the case of *E. coli* (26.9–44.3%), *P. aeruginosa* (37.8–46.3%), *B. subtilis* (44.6–71.5%) and *S. aureus* (53.8–74.9%) (Table 3). (Color figure online)

CD conformational analysis of E2EM-lin in the presence of SUVs formed from either DMPG, CL or DMPE at varying pH was undertaken (Supplementary Fig. 2) and at pH 7, the α-helical structure of the peptide ranged from 42.9% to 61.8% (Table 3). The levels of this α-helical structure were enhanced at pH 8 (54.8–68.3%, Table 3) but reduced at pH 6 (41.0–53.8%, Table 3). Statistical analysis showed that there was a significant difference between the levels of α-helicity possessed by the peptide at pH 6, 7 and pH 8 in the case of membranes formed from DMPG (*F*_2,8_ = 327.2; *p* = 0.000), DMPE (*F*_2,8_ = 164.3; *p* = 0.000) and CL (*F*_2,8_ = 107.9; *p* = 0.000). Corresponding experiments on E2EM-lin were performed with SUVs mimetic of membranes from *S. aureus*, *B. subtilis*, *E. coli* and *P. aeruginosa* (Fig. 2) and at pH 7, E2EM-lin was predominantly α-helical (41.0–63.1%, Table 3). These levels of α-helicity were enhanced at pH 8 (48.3–74.9%, Table 3) and reduced at pH 6 (31.0–53.8%, Table 3). Statistical analysis showed that there was a significant difference between the levels of α-helicity possessed by the peptide at pH 6, pH 7 and pH 8 in the case of SUVs mimetic of membranes of *S. aureus* (*F*_2,8_ = 190.6; *p* = 0.000), *B. subtilis* (*F*_2,8_ = 113.9; *p* = 0.000), *E. coli,* (*F*_2,8_ = 63.7; *p* = 0.000) and *P. aeruginosa* (*F*_2,8_ = 16.3; *p* = 0.004).

**Table 3:** The effect of pH on the conformation of E2EM-lin in the presence of bacterial lipids and membranes

SUVs	Levels of α-helicity (%)		
	pH 6	pH 7	pH 8
Pure lipids			
DMPG	53.8	61.8	68.3
CL	50.2	59.6	67.6
DMPE	41.0	42.9	54.8
Bacterial membrane mimics			
* S. aureus*	53.8	63.1	74.9
* B. subtilis*	44.6	62.1	71.5
* E. coli*	33.8	41.7	49.3
* P. aeruginosa*	31.0	41.0	48.3

This table shows the effect of changing pH on the levels of α-helicity adopted by E2EM-lin in the presence of SUVs formed from either the individual lipids, DMPG, CL or DMPE, or lipid mimics of membranes from *B. subtilis*, *S. aureus*, *E. coli* and *P. aeruginosa* (Table 2). The data are the mean of five replicates

In combination, the data presented in Table 3 clearly show that E2EM-lin has a general, pH dependent ability, to adopt an α-helical structure in the presence of membranes, which is maximal under alkaline conditions (Table 3). Moreover, these data showed that at as pH was changed from pH 7 to either pH 8 or pH 6, the levels of α-helicity adopted by the peptide maintained their rank order (Table 3). In the case of bacterial membrane mimics, the rank order was *S. aureus* > *B. subtilis* > *E. coli* > *P. aeruginosa*, clearly indicating that the peptide adopts higher levels of α-helicity in the presence of membranes from Gram-positive bacteria, as compared to those from Gram-negative bacteria. In the case of pure lipids, this rank order was DMPG > CL > DMPE, clearly showing that E2EM-lin adopts higher levels of α-helicity in the presence of anionic membranes, as compared to zwitterionic membranes. Comparison of these rank orders strongly suggested that the adoption of α-helical structure by E2EM-lin is related to differences in the lipid compositions of bacterial membranes. PG and CL are the major lipids in membranes from Gram-positive bacteria, such as *S. aureus* and *B. subtilis*, whereas PE is the predominant lipid in membranes of Gram-negative organisms, such as *E. coli* and *P. aeruginosa* (Table 2) [55, 56].

### The effect of pH on the ability of E2EM-lin to penetrate and lyse bacterial membranes

The ability of E2EM-lin to interact with bacterial membranes at varying pH was investigated by assessing the ability of the peptide to penetrate monolayers (Supplementary Fig. 3) and lyse SUVs formed from pure lipids (Table 2). The use of Langmuir troughs showed that at pH 7, E2EM-lin penetrated monolayers formed from either DMPG, DMPE or CL, and induced maximal surface pressures in these monolayers that ranged between 2.4 mN m^−1^ and 6.9 mN m^−1^ (Table 4A). These surface pressures were enhanced at pH 8 (3.0–9.8 mN m^−1^, Table 4A), but reduced at pH 6 (1.0–4.7 mN m^−1^, Table 4A), and statistical analysis indicated significant differences between the maximum surface pressure changes induced by the peptide at pH 8 as compared to pH 6 and pH 7 in the case of DMPG (*F*_2,8_ = 5.0; *p* = 0.05), CL (*F*_2,8_ = 10.5; *p* = 0.01) and DMPE (*F*_2,8_ = 10.3; *p* = 0.01). Using SUVs formed from either DMPG, DMPE or CL showed that at pH 7, the peptide induced lysis of these SUVs with levels that ranged between 22.6% and 37.0% (Table 4B) and were enhanced at pH 8 (37.0–51.0%, Table 4B), but reduced at pH 6 (17.0–29.0%, Table 4B). Statistical analysis, showed that there was a significant difference between the levels of lysis exhibited by E2EM-lin at pH 6, 7 and pH 8 in the case of membranes formed from DMPG (*F*_2,8_ = 43; *p* = 0.000), DMPE (*F*_2,8_ = 16.2; *p* = 0.004) and CL (*F*_2,8_ = 9.5; *p* = 0.002).

**Table 4:** The effect of pH on the ability of E2EM-lin to penetrate and lyse bacterial membranes

A. The penetration of lipid monolayers by E2EM-lin			
Lipid monolayers	Change in π (mN m^−1^) induced by E2EM-lin		
	pH 6	pH 7	pH 8
Pure lipids			
DMPG	4.7	6.9	9.8
CL	1.0	2.4	3.0
DMPE	3.1	4.0	4.7
Bacterial membrane mimics			
* S. aureus*	5.9	7.5	9.9
* B. subtilis*	4.5	6.7	9.0
* E. coli*	2.9	4.3	5.2
* P. aeruginosa*	1.3	3.9	4.8

B. The lysis of lipid SUVs by E2EM-lin			
SUVs	Levels of lysis (%) induced by E2EM-lin		
	pH 6	pH 7	pH 8
Pure lipids			
DMPG	26.0	52.7	63.7
CL	17.0	22.6	27.0
DMPE	24.0	33.0	40.0
Bacterial membrane mimics			
* S. aureus*	34.0	60.2	66.5
* B. subtilis*	32.1	57.0	63.1
* E. coli*	27.1	38.0	42.2
* P. aeruginosa*	21.9	35.0	39.9

This table shows the effect of changing pH on the ability of E2EM-lin to penetrate monolayers (A) and lyse SUVs (B) formed from either pure lipids or lipid mimics of bacterial membranes (Table 2). In each case, the data are the mean of five replicates

**Fig. 3:**
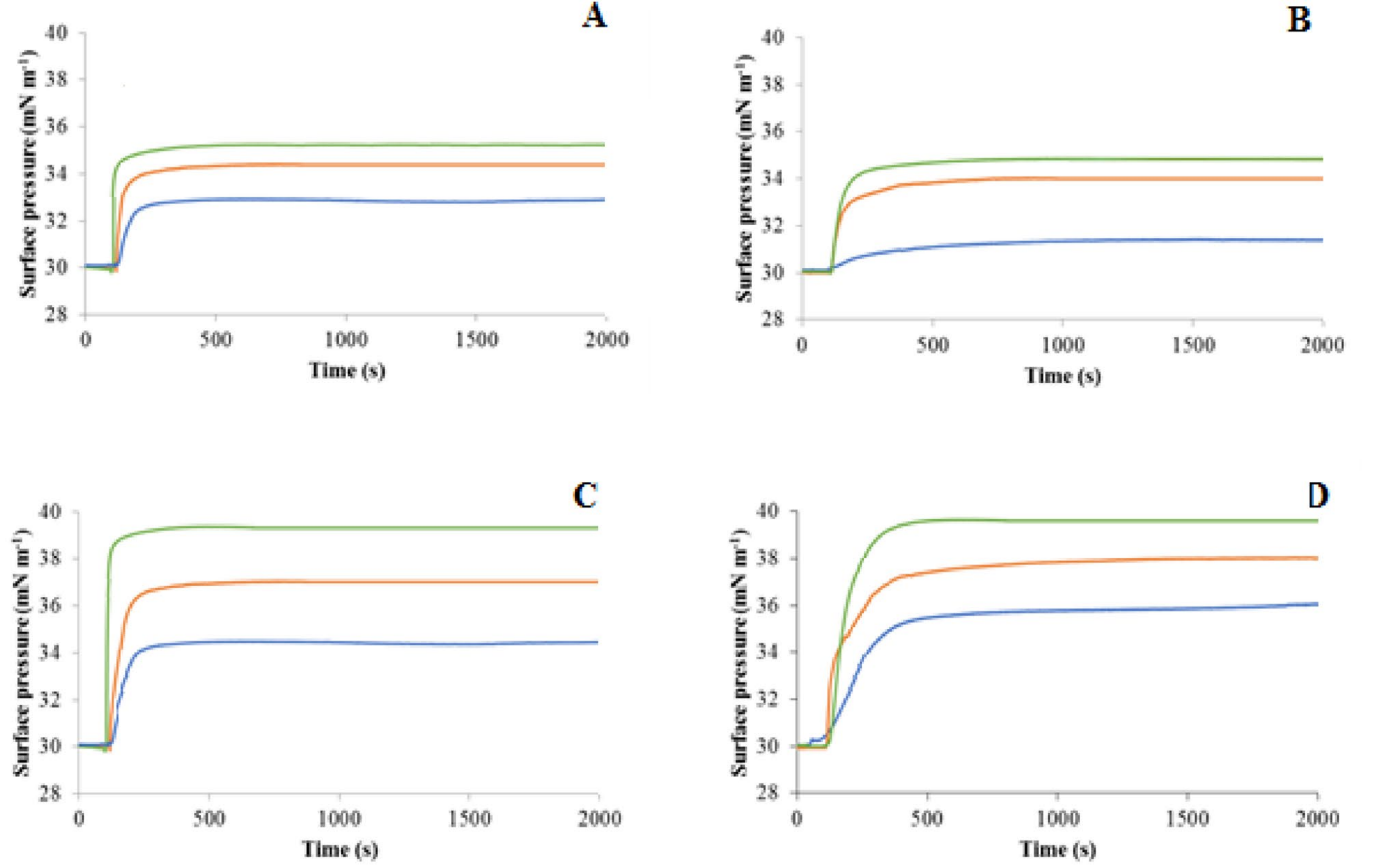
The effect of pH on the ability of E2EM-lin to penetrate and lyse bacterial membranes. This figure shows the effect of changing pH on the interactions of E2EM-lin with lipid monolayers mimetic of bacterial membranes. These interactions are represented by the maximal surface pressure changes induced by the peptide in the case of monolayers mimetic of membranes from *E. coli* (**A**), *P. aeruginosa* (**B**), *B. subtilis* (**C**) and *S. aureus* (**D**) at pH 6 (Blue), 7 (Orange) and 8 (Green). Data derived from these charts showed that the interaction of E2EM-lin with these monolayers were enhanced as pH increased from pH 6 to pH 8 in the case of *E. coli* (2.9–5.2 mN m^−1^), *P. aeruginosa* (1.3–4.8 mN m^−1^), *B. subtilis* (4.5–9.2 mN m^−1^) and *S. aureus* (5.9–9.9 mN m^−1^) (Table 4A). (Color figure online)

The ability of E2EM-lin to interact with bacterial membranes at varying pH was also investigated by assessing the ability of the peptide to penetrate monolayers (Fig. 3) and lyse SUVs formed from lipid mixtures mimetic of bacterial membranes (Table 2). The use of Langmuir troughs showed that at pH 7, E2EM-lin penetrated monolayers mimetic of membranes from either *S. aureus*, *B. subtilis*, *E. coli* or *P. aeruginosa* and induced maximal surface pressures that ranged between 3.9 mN m^−1^ and 7.5 mN m^−1^ (Table 4A). These surface pressure changes were enhanced at pH 8 (4.8–9.9 mN m^−1^, Table 4A), but reduced at pH 6 (1.3–5.9 mN m^−1^, Table 4A), and statistical analysis indicated significant differences between the maximum surface pressure changes induced by the peptide at pH 8 as compared to pH 6 and pH 7 in the case of *S. aureus* (*F*_2,8_ = 10.3; *p* = 0.01), *B. subtilis* (*F*_2,8_ = 11.9; *p* = 0.008), *E. coli* (*F*_2,8_ = 5.7; *p* = 0.04) and *P. aeruginosa* (*F*_2,8_ = 7.7; *p* = 0.02). Using SUVs mimetic of membranes from either *S. aureus*, *B. subtilis*, *E. coli* or *P. aeruginosa*, showed that at pH 7, E2EM-lin induced levels of lysis that ranged between 35.0% and 60.2% (Table 4B). These levels of lysis were enhanced at pH 8 (39.9–66.5%, Table 4B), but reduced at pH 6 (24.9–34.0%, Table 4B) and statistical analysis showed that there was a significant difference between the levels of lysis exhibited by E2EM-lin at pH 6, 7 and pH 8 in the case of SUVs mimicking membranes of *S. aureus* (*F*_2,8_ = 713.9; *p* = 0.000), *B. subtilis* (*F*_2,8_ = 171.9; *p* = 0.000), *E. coli* (*F*_2,8_ = 44; *p* = 0.000) and *P. aeruginosa* (*F*_2,8_ = 44.3; *p* = 0.004).

In combination, the data presented in Table 4 clearly showed that E2EM-lin has a general, pH dependent ability, to penetrate and lyse membranes, which is maximal under alkaline conditions (Table 4). Moreover, these data also showed that as pH is changed from pH 7 to either pH 8 or pH 6, the levels of membrane penetration and lysis displayed by the peptide maintained their rank order (Table 4). In the case of bacterial membrane mimics, the rank order was *S. aureus* > *B. subtilis* > *E. coli* > *P. aeruginosa*, clearly indicating that the peptide has an enhanced capacity to penetrate and lyse membranes of Gram-positive bacteria, as compared to those from Gram-negative bacteria. In the case of pure lipids, the rank order was DMPG > DMPE > CL (Table 4), which parallels that observed for the induction of α-helical structure in E2EM-lin by the first two of these lipids (Table 3). imilar correlation of rank orders was reported by previous work on E2EM-lin and, in combination, these data suggest that the peptide penetrates and lyses bacterial membranes via mechanisms involving the adoption of α-helical structure that are driven by PG in the case of membranes from Gram-positive bacteria and PE in the case of those of Gram-negative bacteria (Table 4A, B) [37, 57].

E2EM-lin showed the lowest levels of penetration and membrane (Table 4) observed for the at each pH studied although adopting levels of α-helicity that were comparable to those recorded in the case of PG membranes (Table 3). In combination, these data suggest that, although CL is able to promote stabilization of the peptide’s α-helical structure and membrane binding, the lipid does not promote the levels of partitioning required for membranolysis by E2EM-lin across the pH range studied. Clearly, either some property of CL can inhibit the ability of the peptide to partition into membranes or the lipid can promote the antibacterial activity of E2EM-lin by mechanisms other than those involving direct membrane lysis. Based on these results it seems unlikely that CL driven mechanisms make a major contribution to the membranolytic activity of E2EM-lin in the case of either Gram-positive or Gram-negative bacteria.

### The effect of pH on the thermodynamic stability of E2EM-lin interactions with bacterial membranes

Langmuir troughs were used to generate compression isotherms and investigate the effect of varying pH on the thermodynamic stability of E2EM-lin interactions with monolayers mimetic of membranes from *S. aureus*, *B. subtilis*, *E. coli* and *P. aeruginosa* (Supplementary Figs. 3 and 4). Isotherm data derived from supplementary Figs. 3 and 4 were used to calculate Δ*G*_mix_ for these monolayers in the absence and presence E2EM-lin at 20 mN m^−1^ and across the pH range pH 6–8 (Table 5). In the absence of the peptide, these monolayer mimics of bacterial membranes were thermodynamically stable in every case, with values of Δ*G*_mix_ < 0. However, as pH was increased from pH 6 to pH 8, Δ*G*_mix_ became progressively less negative, indicating that the thermodynamic stability of these monolayers was decreased by alkaline conditions (Table 5). Statistical analysis indicated that in the absence of E2EM-lin, there were significant differences between the values of Δ*G*_mix_ observed at pH 8 as compared to pH 6 and pH 7 (Table 5) in the case of monolayers representing membranes of *S. aureus* (*F*_2,8_ = 6252.9; *p* = 0.000), *B. subtilis* (*F*_2,8_ = 40,970; *p* = 0.000), *E. coli* (*F*_2,8_ = 299.4; *p* = 0.000) and *P. aeruginosa* (F_2,8_ = 3160; *p* = 0.000). In contrast, in the presence of the peptide, monolayers mimetic of membranes from *S. aureus*, *B. subtilis*, *E. coli* and *P. aeruginosa* exhibited values of Δ*G*_mix_ > 0, indicating that in every case, these monolayers are thermodynamically unstable (Table 5). However, as pH was increased from pH 6 to pH 8, Δ*G*_mix_ became progressively less positive, indicating that the thermodynamic instability of these monolayers due to the presence of E2EM-lin was reduced at higher pH (Table 5). Statistical analysis indicated that in the presence of the peptide, there were significant differences between the values of Δ*G*_mix_ at pH 8 as compared to pH 6 and pH 7 (Table 5) for lipid monolayers representing *S. aureus* (*F*_2,8_ = 13,905; *p* = 0.000), *B. subtilis* (*F*_2,8_ = 12,638; *p* = 0.000), *E. coli* (*F*_2,8_ = 2741; *p* = 0.000) and *P. aeruginosa* (*F*_2,8_ = 15,975; *p* = 0.000).

**Table 5:** The effects of changing pH on Δ*G*_mix_ for bacterial membranes

Bacterial membrane mimics	Δ*G*_mix_ (kJ mol^−1^)		
	pH 6	pH 7	pH 8
In the absence of E2EM-lin			
* B. subtilis*	− 40.6	− 26.3	− 2.3
* S. aureus*	− 38.0	− 31.9	− 12.1
* E. coli*	− 16.1	− 6.3	− 4.0
* P. aeruginosa*	− 40.1	− 39.6	− 29.9
In the presence of E2EM-lin			
* B. subtilis*	63.5	44.6	18.7
* S. aureus*	58.1	50.1	18.3
* E. coli*	97.3	78.2	29.0
* P. aeruginosa*	79.6	62.7	40.1

This table shows the effect of changing pH on Δ*G*_mix_ for lipid mimics of bacterial membranes at 20 mN m^−1^ in the absence and presence of E2EM-lin. Values of Δ*G*_mix_ < 0 indicate thermodynamic stability and those > 0 show thermodynamic instability and in each case, the data are the mean of five replicates [50, 51]

In combination, these results showed that E2EM-lin has a pH dependent, thermodynamically destabilizing effect on bacterial membranes, that was minimal under alkaline conditions, indicating that the relative effect of E2EM-lin was to thermodynamically stabilize these membranes as pH was increased (Table 5). Taken with the results of recent studies on the peptide, these observations suggested that pH dependent increases in the levels of α-helicity possessed by E2EM-lin promoted decreases in the lipid packing properties of bacterial membranes [37]. These lipid packing properties included their lateral pressure, rigidity and lipid packing density, and reductions in the levels of these properties would be consistent with the higher levels of membrane penetration and membrane lysis observed for E2EM-lin under alkaline conditions (Table 4A, B).

## Discussion

**Fig. 4:**
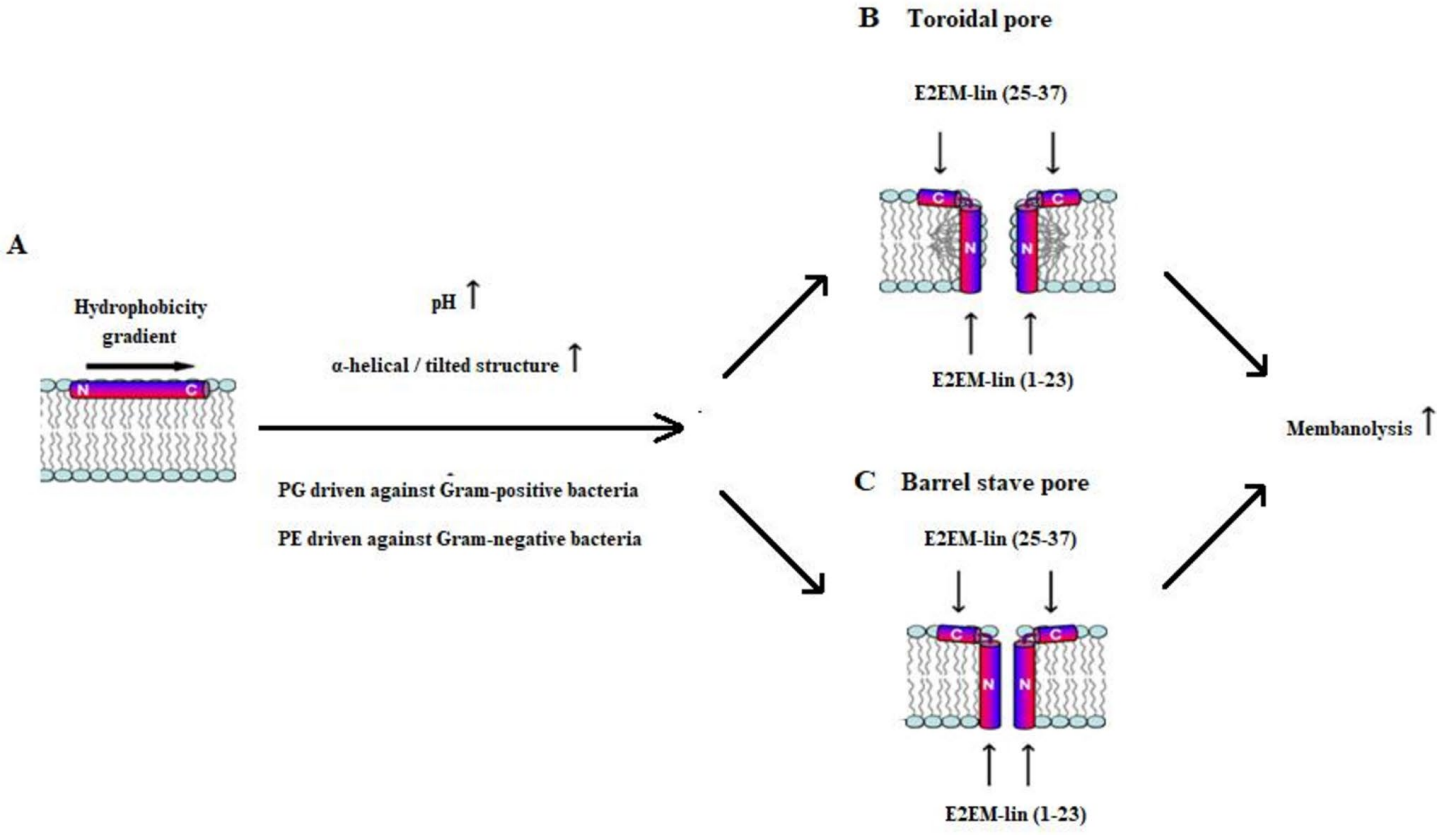
A model for the pH dependent, membranolytic antibacterial action of E2EM-lin. This figure is revised from previous work [37, 53] and shows a schematic representation of the pH dependent antimicrobial action proposed for E2EM-lin. Initially, the peptide interacts with the bacterial membrane surface and forms α-helical structure (represented as cylinders) with a hydrophobic surface (red) and a hydrophilic surface (blue) (**A**). The α-helical structure formed by E2EM-lin (1–23) possesses a hydrophobicity gradient and the levels of this structure are enhanced by increasing pH (**A**). This tilted segment then promotes pore formation by E2EM-lin via membrane insertion and the adoption of a transmembrane orientation, which is stabilized by the surface interactions of E2EM-lin (25–37) (**B**, **C**). Potentially, E2EM-lin can form a toroidal pore (**B**) or a barrel stave pore (**C**) and the major difference between these pore types is that in the former pore, the membrane leaflets deform to allow the lipid head-group region to remain in contact with the hydrophilic face of the E2EM membrane spanning region, which is not observed in the latter pore [54]. In both cases, increasing pH promotes higher levels of membranolysis, which are maximal under alkaline conditions (**B**, **C**). For clarity, two monomers of E2EM-lin are shown in this pore forming process but it has been predicted that the involvement of higher order oligomers of the peptide are probable [57]. (Color figure online)

In the present work, it has been shown that E2EM-lin from *G. emeljanovi* has potent activity against *S. aureus* and *B. subtilis* at levels suitable for therapeutic development (≤ 6.25 μM, Table 2). However, the peptide was *circa* 30-fold times less effective against *E. coli* and *P. aeruginosa* (≥ 75.0 μM, Table 2) and in combination, these results clearly show that E2EM-lin has a strong preference for Gram-positive bacteria, which is consistent with previous studies [37, 53, 58]. The antibacterial action of E2EM-lin under neutral conditions was investigated and using *S. aureus* as an example, the peptide was found to be predominantly α-helical in the presence of the organism’s membranes (63.1%, Table 3) and showed a strong ability to penetrate (∆π = 7.5 mN m^−1^, Table 4A) and lyse these membranes (60.2%, Table 4B). Similar results were observed for each of the remaining bacteria studied here (Tables 3 and 4) and in combination, these data clearly suggested that a major driver in the antibacterial action of E2EM-lin is the adoption of lipid interactive α-helical structure (Fig. 1) that promote membranolytic mechanisms. These observations strongly supported a recently presented model for the antimicrobial action of the peptide, which involves pore formation and is depicted in Fig. 4.

Changes to pH were found to strongly influence the action of E2EM-lin against *S. aureus*, with acid conditions reducing the α-helicity of the peptide in the presence of the organism’s membranes (53.8%, Table 3) and decreasing its capacity to penetrate (∆π = 5.9 mN m^−1^, Table 4A) and lyse these membranes (34.0%, Table 4B). In contrast, moving pH to alkaline conditions enhanced the α-helicity of E2EM-lin the presence of *S. aureus* membranes (74.9%, Table 3) and increased the peptide’s capacity to penetrate (∆π = 9.9 mN m^−1^, Table 4A) and lyse them (66.5%, Table 4B). Similar pH dependent changes to these E2EM-lin properties were observed for the remaining bacteria studied here, and in combination, these data showed that alkaline pH promoted the membranolytic, antibacterial action of E2EM-lin by increasing its lipid interactive, α-helical contributions.

Thermodynamic data suggested that the partitioning of increasingly α-helical E2EM-lin into the lipid matrix of these membranes effectively decreased their rigidity and lipid packing density as pH was increased (Table 5) [37]. This effect would seem to help explain the relative susceptibility shown by membranes of *B. subtilis*, *S. aureus*, *E. coli* and *P. aeruginosa* to the lytic action of E2EM-lin under alkaline conditions and their relative resistance to this action under acid conditions. The individual lipids forming the lipid matrix of these target bacterial membranes, PG, CL and PE, were each found to promote the adoption of membrane interactive α-helical structure by the peptide (Table 3), although their contribution to the membranolytic action of E2EM-lin varied widely (Table 4).

PG is one of the predominant lipid species in the strongly anionic membranes of Gram-positive bacteria [55, 56] and appeared to be the major driver of the action of E2EM-lin against these organisms. In response to increasing pH, PG induced the highest levels of α-helical structure (≥ 53.8%, Table 3), membrane penetration (≥ 4.7 mN m^−1^, Table 4A) and membrane lysis (≥ 29.0%, Table 4B) recorded for E2EM-lin, which is consistent with the generally high affinity of cationic AMPs for anionic lipid [59]. These observations suggest that pH dependent, PG driven increases in the α-helicity of E2EM-lin enhance its levels of amphiphilicity (Table 4, Fig. 2). These elevated levels of amphiphilic structure would then facilitate higher levels of electrostatic interaction with PG head groups and concomitant, increased levels of hydrophobic association with the acyl chain region of membranes under alkaline conditions (Table 4, Fig. 3). Indeed, it is noticeable that the lysine residues of E2EM-lin are distributed along the full length of the peptide and it seems likely that pH dependent increases in the α-helicity of E2EM-lin would enhance the recruitment and availability of these residues to both target PG and participate in membrane interactions ((Fig. 1C, D). In combination, these observations clearly suggest that pH dependent, PG-mediated increases in amphiphilic α-helical structure make a major contribution to the activity of E2EM-lin against Gram-positive bacteria. Given the low levels of PG in the membranes of Gram-negative bacteria, it seems likely that similar mechanisms could also make a minor contribution to the action of E2EM-lin against these organisms (Table 2).

CL is the other predominant lipid species found in the membranes of Gram-positive bacteria [55, 56] and E2EM-lin showed interactions with membranes formed from this lipid that differed strongly to those exhibited by the peptide with PG (Tables 3, 4A, B). In response to increasing pH, although CL induced levels of α-helical structure in E2EM-lin that were comparable to those induced by PG (≥ 50.2%, Table 3), the lipid promoted the lowest levels of membrane penetration (≤ 3.0 mN m^−1^, Table 4A) and membrane lysis (≤ 26.0%, Table 4B) recorded for E2EM-lin. This peptide clearly has some capacity to penetrate and lyse membranes formed from CL, but these levels of interaction are greatly reduced compared to those observed with PG (Table 4, Fig. 3). The reasons for these observations are unclear but one potential explanation is that the binding of E2EM-lin to CL membranes facilitates the adoption of α-helical structure, and the strong affinity of these binding events restricts, but does not abolish, access of the peptide to these membranes. A strong affinity for CL membranes could be facilitated by the characteristics of the lipid’s headgroup, which exists as a dimeric structure comprising two linked phosphate moieties that give membranes formed from the lipid a high density of negative surface charge [60–62]. Given the distribution of positive charges along the length of E2EM-lin, it seems likely that these residues promote binding of the peptide to CL membranes. However, the far end region of the peptide’s predicted N-terminal, tilted segment lacks these residues and we speculate that this region of E2EM-lin may be able to access CL membranes and contribute to the low levels of pH dependent, lysis observed for the peptide (Figs. 1B and 1C). In combination, these observations clearly suggest that pH-mediated high affinity interactions with CL headgroups make a major contribution to the activity of E2EM-lin against Gram-negative bacteria. Based on the low levels of CL in the membranes of Gram-positive bacteria, it seems likely that similar mechanisms could also make a minor contribution to the action of E2EM-lin against these organisms (Table 2).

PE is the predominant lipid in the membranes of Gram-negative bacteria [55, 56] and appeared to be the primary driver of the action of E2EM-lin against these organisms. In response to increasing pH, PE induced levels of α-helical structure (≥ 41.0%, Table 3), membrane penetration (≥ 3.1 mN m^−1^, Table 4A) and membrane lysis (≥ 24.0%, Table 4B). However, the magnitude of these E2EM-lin properties was reduced by around a quarter to a half, as compared to those observed in the case of PG, clearly suggesting that the peptide has a relatively lower affinity for PE (Tables 3, 4A, B), which is typical of AMPs with a preference for Gram-positive bacteria [63]. In combination, these observations suggest that pH dependent, PE driven increases in the α-helicity of E2EM-lin enhance the hydrophobicity of the peptide thereby promoting higher levels of interaction with the acyl chain region of membranes under alkaline conditions. These observations also clearly suggest that these PE-mediated mechanisms make a major contribution to the activity of E2EM-lin against Gram-positive bacteria. Given the low occurrence of PE in the membranes of Gram-negative bacteria, it seems likely that similar mechanisms could also make a minor contribution to the action of E2EM-lin against these organisms (Table 2).

It has previously been suggested that pH related changes to the structural characteristics of lipid could contribute to the antibacterial action of E2EM-lin peptide [9] and it is well established that the headgroup charge and morphology of these molecules are primary determinants in the membrane interactions of AMPs [64]. Across the pH range studied here, PG maintains a charge of -1 whilst PE shows no change to its zwitterionic charge, rendering it unlikely that pH related charge effects to either lipid will make a major, direct contribution to the pH dependent membranolytic activity of E2EM-lin [64, 65]. In contrast, there is the possibility that pH related changes to the structural characteristics of CL could contribute to the membrane interactions of E2EM-lin, given that across the pH range studied here, the charge on the lipid increases from − 1 to − 2 [60, 66]. This pH dependent charge effect could enhance the initial electrostatic interaction between E2EM-lin and bacterial membranes at higher pH, thereby helping to compensate for the decreased net positive charge of the peptide under these pH conditions. Clearly, this pH dependent charge effect could also contribute to the increased binding affinity of E2EM-lin for bacterial membranes under alkaline conditions (Table 4, Fig. 3).

In relation to lipid morphology, PG, has a strong preference for the lamellar phase and PE possesses a cone shaped morphology that are both effectively maintained across the pH range studied here [64, 65]. These observations clearly suggest that pH dependent, packing effects associated with either lipid will not make a major, direct contribution to the pH dependent membranolytic activity of E2EM-lin. In the case of CL, although cone shaped, the lipid effectively behaves as a lamellar lipid, across the pH range studied here due to intermolecular, electrostatic repulsion effects between the negative charges of its headgroups [60, 66]. These effects promote looser lipid packing in CL membranes at higher pH, which could enhance the ability of E2EM-lin structure to access to these membranes and thereby help explain the pH dependency of CL membrane lysis by the peptide (Table 4, Fig. 3). In combination, these observations clearly suggest that changes to the intrinsic properties of CL have the potential to contribute to the pH dependent membranolytic activity of E2EM-lin against both Gram-positive and Gram-negative bacteria (Table 2).

Studies on the interaction of E2EM-lin with individual bacterial lipids clearly suggested that the ability to engage in electrostatic associations played a major role in the selectivity and efficacy of the peptide’s pH dependent antimicrobial action. The effect of pH on the net charge of E2EM-lin was determined, which showed that this charge drops from + 4 at acid pH to + 3 at alkaline pH, which would clearly decrease the ability of the peptide to engage in electrostatic interactions with these membranes with increasing pH. However, it seems likely that this effect would be compensated for, at least in part, by the enhanced ability of CL to contribute to these interactions through the elevation of its negative charge by increasing pH. The major effect of a decreased positive charge with increasing pH on E2EM-lin would seem to be to effectively render the peptide more hydrophobic, thereby promoting the tilted, membranolytic action of the peptide. (Fig. 4B, C). Currently, it is believed that this action involves the ability of the peptide to form either barrel-stave pores or toroidal pores [37, 53], although the latter pore type is most consistent with experimental data [53]. In either case, pH-mediated enhancement of membranolytic, α-helical structure possessed by the peptide could support its action against both Gram-positive and Gram-negative bacteria by a number of mechanism [37]. As a major example, the induction of increased levels of α-helical structure in E2EM-lin (1–23) by alkaline pH could increase the levels of its tilted structure (Fig. 4A). Increased levels of this tilted structure would then drive the enhanced capacity for bacterial membrane penetration and lysis observed for E2EM-lin under these pH conditions (Fig. 4B, C, Table 4). It is well established that the oblique mode of membrane penetration utilized by tilted peptides can lead to a range of membrane destabilizing effects [40, 54], including the promotion of pore formation [67]. Indeed, E2EM-lin (1–23) forms the transmembrane component of pores formed by the parent peptide (Fig. 4B, C) and it is well established that tilted characteristics can also promote peptide–peptide interactions that assist in the assembly and stabilization of pores [38]. In addition to E2EM-lin (1–23), the induction of higher levels of α-helical structure in E2EM-lin (25–37) under alkaline conditions could promote the efficiency of pore formation by E2EM-lin. E2EM-lin (25–37) is believed to support pore construction by engaging in surface interactions with target bacterial membranes that anchor and stabilize the transmembrane orientation of E2EM-lin (1–23) (Fig. 4B and C) [37]. No tilted structure appeared to be formed by E2EM-lin (25–37) and the distribution of hydrophobicity along the α-helical long axis of this segment was approximately constant (Fig. 1), indicating that it could be expected to orientate parallel to the membrane surface, consistent with its anchoring function [68]. Enhanced levels of α-helicity in E2EM-lin (25–37) under alkaline conditions could therefore promote the efficacy of the segment’s anchoring function and the membranolytic antibacterial action of the parent peptide.

In conclusion, the present study has shown that E2EM-lin possesses pH dependent, membranolytic antibacterial activity with an alkaline optimum and a preference for action against Gram-positive bacteria over Gram-negative bacteria. This membranolytic activity appeared to depend upon the level of positive charge carried by E2EM-lin and the induction of lipid interactive tilted and α-helical structure, which was driven by PG in the case of Gram-positive bacteria and PE in that of Gram-negative bacteria. In contrast, the induction of α-helical structure by CL appeared to reduce the membranolytic activity of E2EM-lin by promoting high affinity interactions with the lipid that restricted the access of the peptide to membranes. Countering this inhibitory effect, pH dependent changes to the intrinsic structural characteristics of CL showed the potential to enhance the initial membrane binding and membranolytic activity of E2EM-lin. These data were used to generate a novel pore-forming model for the membranolytic activity of E2EM-lin, which would appear to be the first, major reported instance of pH dependent AMPs with alkaline optima using tilted structure to drive a pore-forming process (Fig. 4) [36]. The only other major example of pH dependent AMPs using tilted structure to drive their antimicrobial mechanisms would appear to be maximin H5 from *Bombina maxima* (The Yunnan firebelly toad), which has an acid optimum [69]. As described above, there is a clear need for agents to treat diseases and disorders associated with high pH, and taking insights gained here into the antibacterial mechanisms used by E2EM-lin with its lack of haemolytic activity, the peptide has the potential to fulfil this need [57]. For example, E2EM-lin may be suitable for development into a topical antimicrobial agent, typically the treatment of chronic wounds; the alkaline pH of these wounds promotes colonization by a range of pathogenic bacteria, including Gram-positive organisms such as *S. aureus* [70–72]. E2EM-lin could also potentially serve biotechnical applications; for example, as a food preservative; a variety of bacteria including Gram-positive organisms, such as *Brochothrix thermosphacta*, possess tolerance to high pH and act as food spoilage organisms [73, 74].

## Supplementary Information

Below is the link to the electronic supplementary material.

Supplementary file1 (TIFF 70,333 KB)

Supplementary file2 (TIFF 329 KB)

Supplementary file3 (TIFF 327 KB)

Supplementary file4 (TIFF 330 KB)

Supplementary file5 (TIFF 337 KB)

Supplementary file6 (DOCX 18 KB)

## Data Availability

All data generated or analysed during this study are included in this published article and its supplementary information files.
