# Supplementary figures and images for: Linearized esculentin-2EM shows pH dependent antibacterial activity with an alkaline optimum
Source: Mol Cell Biochem. 2021 Jun 6;476(10):3729–44. doi: 10.1007/s11010-021-04181-7 (PMC8382640; doi:10.1007/s11010-021-04181-7)

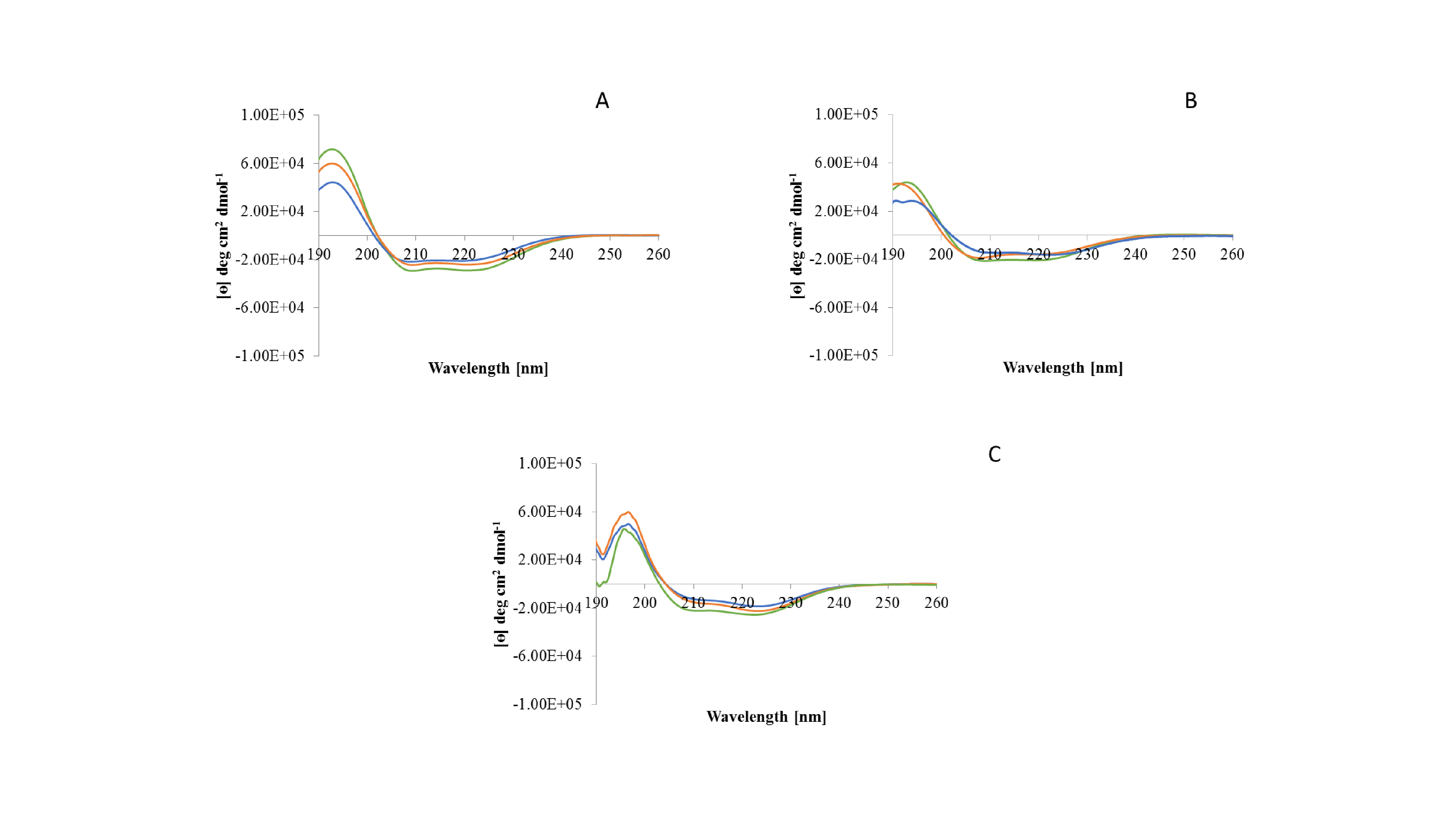

Supplement: Supplementary file 2 — Supplementary file2 (TIFF 329 KB) [file 11010_2021_4181_MOESM2_ESM.tiff]

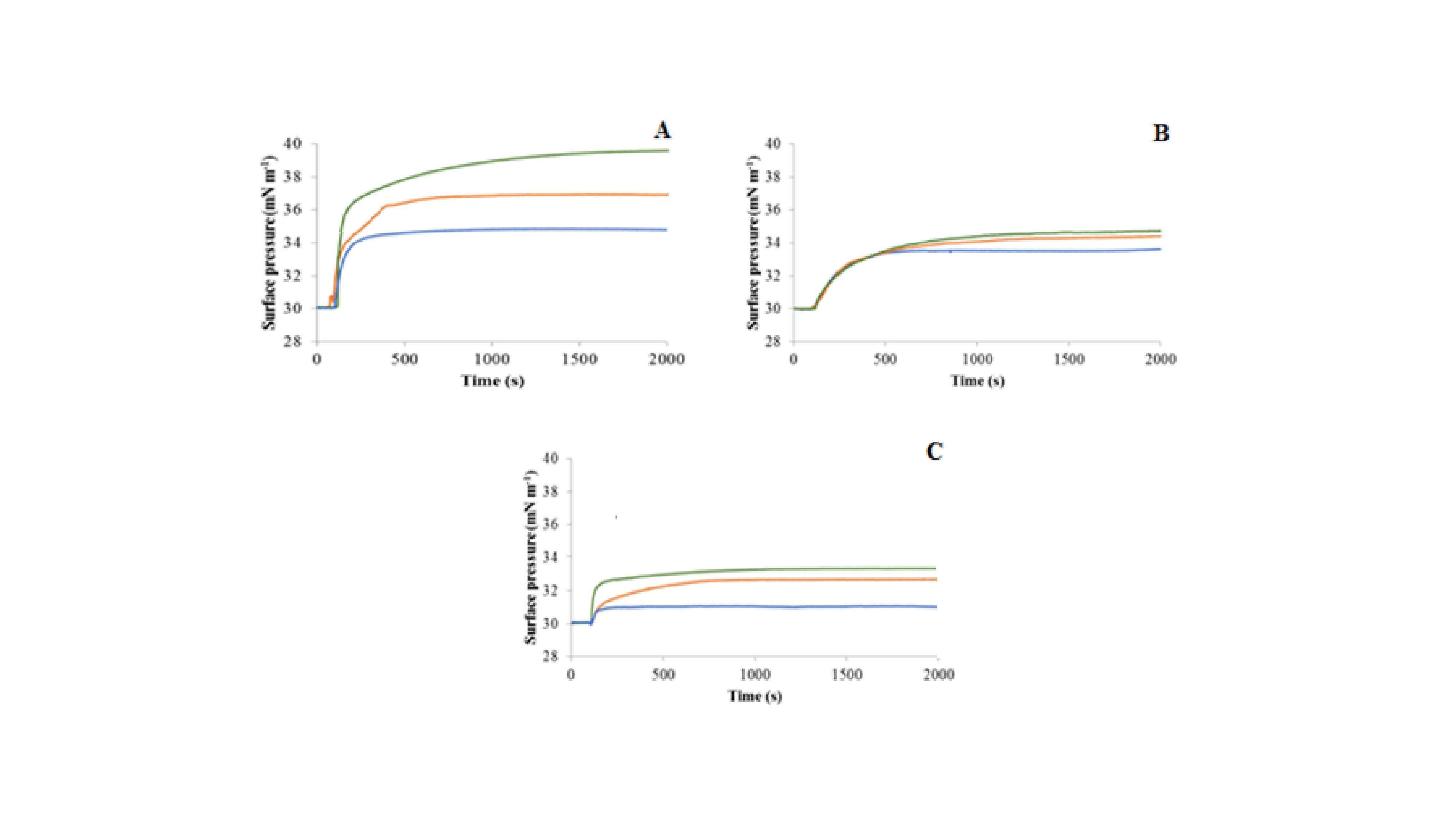

Supplement: Supplementary file 3 — Supplementary file3 (TIFF 327 KB) [file 11010_2021_4181_MOESM3_ESM.tiff]

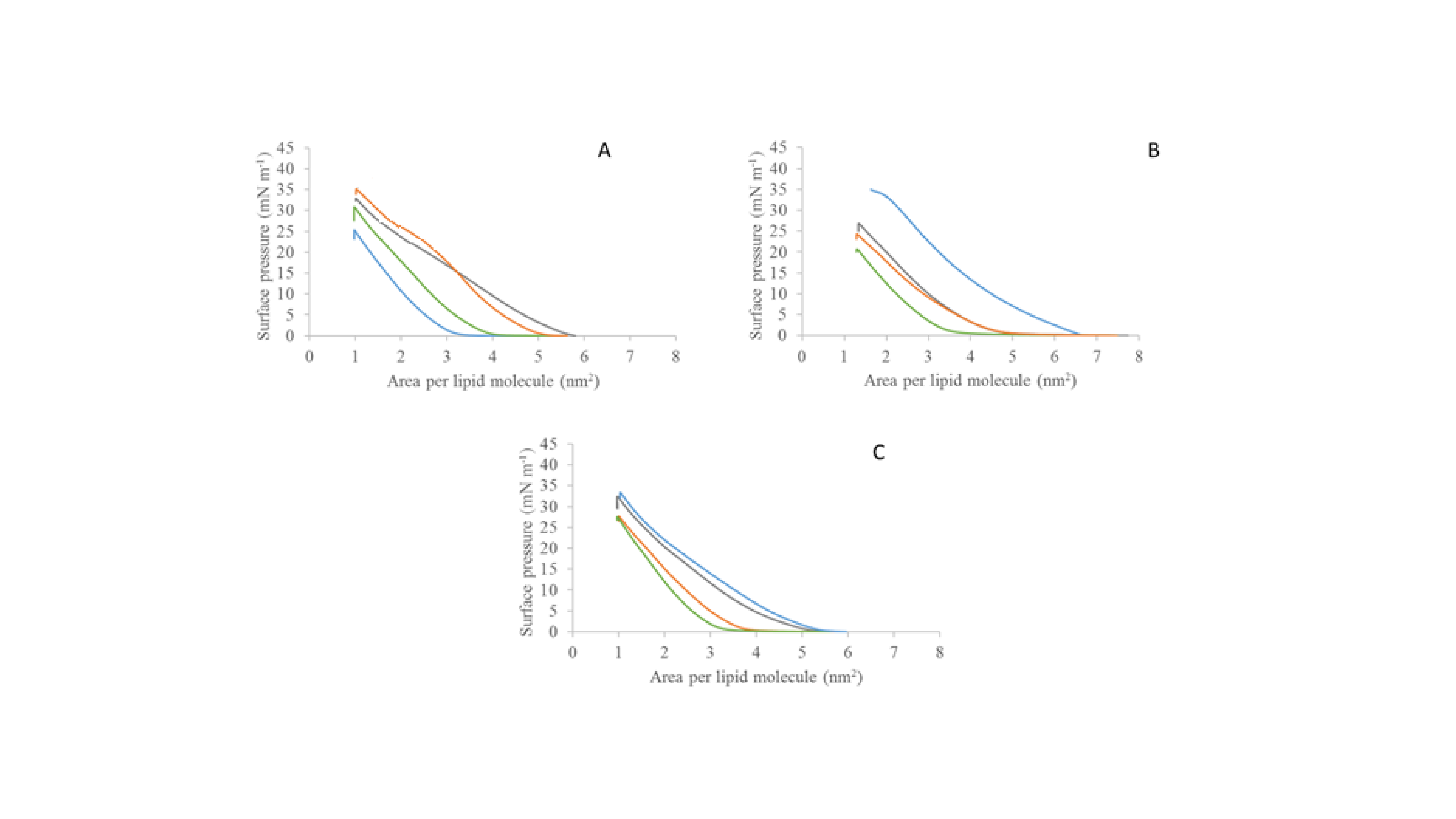

Supplement: Supplementary file 4 — Supplementary file4 (TIFF 330 KB) [file 11010_2021_4181_MOESM4_ESM.tiff]

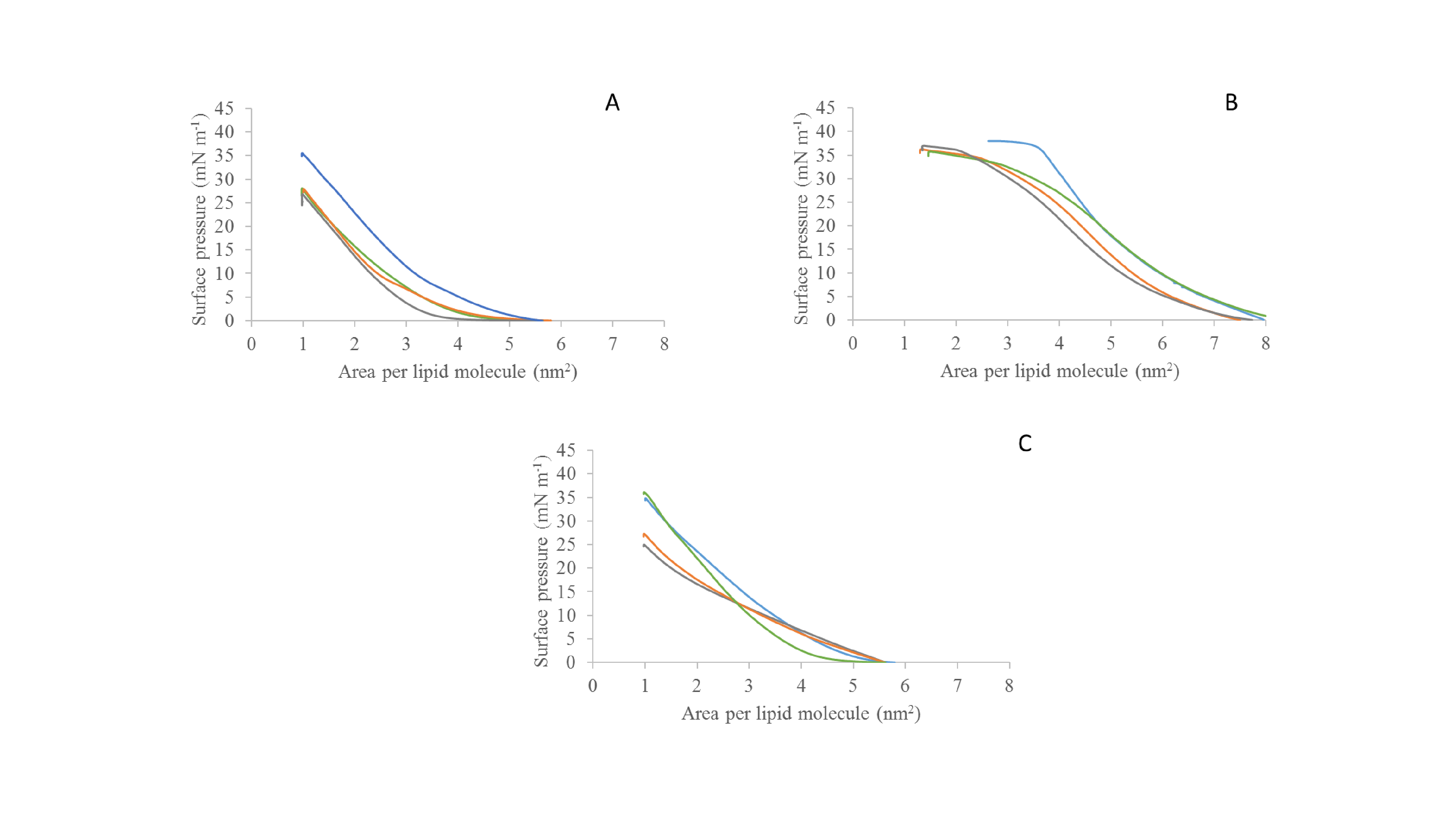

Supplement: Supplementary file 5 — Supplementary file5 (TIFF 337 KB) [file 11010_2021_4181_MOESM5_ESM.tiff]
