# Supplementary material for: Linearized esculentin-2EM shows pH dependent antibacterial activity with an alkaline optimum
Source: Mol Cell Biochem. 2021 Jun 6;476(10):3729–44. doi: 10.1007/s11010-021-04181-7 (PMC8382640; doi:10.1007/s11010-021-04181-7)
Supplement: Supplementary file 6 — Supplementary file6 (DOCX 18 KB) [file 11010_2021_4181_MOESM6_ESM.docx]

**Supplementary data Figure legends**

**Figure 1. Mass spectrometry data for E2EM-lin.** Figures. 1A and 1B, respectively, show the structures of E2EM and E2EM-lin, as predicted by Chemdraw, and Figure. 1C shows a MALDI-TOF time-of-flight mass spectrum of positive ions from E2EM-lin used in this study at a mass of 3747.67, confirming these predictions.

**Figure 2.** **The effect of pH on the conformation of E2EM-lin in the presence of bacterial lipids.** Figure 2 shows the effect of changing pH on the conformational behaviour of E2EM-lin in the presence of SUVs formed from pure bacterial lipids, which were DMPE (**A**), DMPG (**B**) and CL (**C**) at pH 6 (Blue), pH 7 (Orange) and pH 8 (Green). In all cases, these curves possesses minima in the range 210 nm to 224 nm and a maxim around 193 nm, which is typical of α-helical structure [42]. Analysis of these spectra showed that the levels of α-helicity possessed by E2EM-lin were enhanced as pH increased from pH = 6 to pH = 8 in the case of DMPG (53.8% to 68.3%), DMPE (41.0% to 54.0%) and CL (49.7% to 67.6%) (Table 3).

**Figure 3. The effect of pH on the ability of E2EM-lin to penetrate and lyse membranes formed from bacterial lipids.** Figure 3 shows the effect of changing pH on the interactions of E2EM-lin with monolayers formed from pure bacterial lipids, which are represented by the maximal surface pressure changes induced by E2EM-lin in monolayers formed from DMPG (**A**) DMPE (**B**) and CL (**C**) at pH 6 (Blue), 7 (Orange) and 8 (Green). Data derived from these charts showed that the interaction of E2EM-lin with these monolayers was enhanced as pH increased from pH = 6 to pH = 8 in the case of DMPG (4.7 mN m^-1^ to 9.8 mN m^-1^), DMPE (3.1 mN m^-1^to 4.7 mN m^-1^) and CL (1.0 mN m^-1^ to 3.0 mN m^-1^) (Table 4A).

**Figure 4. The effect of pH on the thermodynamic stability of bacterial membranes in the absence of E2EM-lin.** Figure 4 shows the effect of changing pH on the thermodynamic stability of monolayers mimetic of bacterial membranes in the absence of E2EM-lin, which were those representing *E. coli* (Blue), *P. aeruginosa* (Green), *B. subtilis* (Grey) and *S. aureus* (Orange) at pH 6 (**A**), pH 7 (**B**) and pH 8 (**C**). Data derived from these charts showed that these monolayer mimics of bacterial membranes were thermodynamically stable in every case, with values of Δ*G_mix_* < 0. However, as pH was increased from pH 6 to pH 8, Δ*G_mix_* became progressively less negative, indicating that the thermodynamic stability of these monolayers was decreased by alkaline conditions (Table 5).

**Figure 5. The effect of pH on the thermodynamic stability of bacterial membranes in the presence of E2EM-lin.** Figure 5 shows the effect of changing pH on the thermodynamic stability of monolayers mimetic of bacterial membranes in the presence of E2EM-lin, which were those representing *E. coli* (Blue), *P. aeruginosa* (Green), *B. subtilis* (Grey) and *S. aureus* (Orange) at pH 6 (**A**), pH 7 (**B**) and pH 8 (**C**). Data derived from these charts showed that these monolayer mimics of bacterial membranes were thermodynamically unstable in every case, with values of Δ*G_mix_* > 0. However, as pH was increased from pH 6 to pH 8, Δ*G_mix_* became progressively less positive, indicating that the thermodynamic instability of these monolayers was reduced at higher pH (Table 5).
